# A pilot study evaluating the safety and efficacy of En‐bloc holmium laser enucleation of the prostate in patients with a history of radiation therapy or high intensity focused ultrasound for management of organ confined prostate cancer with review of the literature

**DOI:** 10.1002/bco2.70061

**Published:** 2025-07-21

**Authors:** Renil S. Titus, Ansh Bhatia, Joao G. Porto, Jean C. Daher, Adele Raymo, Maggie Meyreles, Archan Khandekar, Aravindh Rathinam, Jonathan Katz, Robert Marcovich, Hemendra N. Shah

**Affiliations:** ^1^ Houston Methodist Hospital Houston Texas USA; ^2^ Department of Interventional Radiology University of Miami Miller School of Medicine Miami Florida USA; ^3^ Desai Sethi Urology Institute University of Miami Miller School of Medicine Miami Florida USA

**Keywords:** bladder outlet obstruction, BPH, complications, HIFU, HoLEP, prostate cancer, radiation therapy

## Abstract

**Objectives:**

To evaluate the safety and efficacy of Holmium Laser Enucleation of the Prostate (HoLEP) in managing patients with a history of organ‐preserving treatments (OPT: Radiation Therapy – RT, High Intensity Focused Ultrasound ‐ HIFU) for Organ‐Confined Prostate Cancer (OC‐PCa).

**Methods:**

We reviewed men undergoing “en‐bloc” HoLEP between July 2017 and December 2023 from our institutional database to identify those with a history of OPT for OC‐PCa (study group). A 1:2 matched‐pair analysis was performed comparing these patients with a control group of men undergoing HoLEP for benign prostatic hyperplasia (BPH) without prior OPT. Demographic, perioperative and postoperative voiding parameters up to 1 year, as well as complications, were compared between groups. Unpaired t‐tests were used for parametric variables and Wilcoxon Rank tests for non‐parametric variables. A p‐value<0.05 was considered statistically significant.

**Results:**

Of 660 patients, 15 had prior OPT before HoLEP. The time between OPT and HoLEP ranged from 3‐month to 12‐year. Associated urethral stricture and/or extensive prostatic calcification were present in five patients. Demographic and preoperative parameters were similar between the groups. However, the study group patient had significantly less resected tissue and higher rate of urinary incontinence at 3‐month. Two patients (13.3%) continued to experience incontinence at 1‐year. There was no clinically significant difference in postoperative improvement in voiding parameters amongst both groups.

**Conclusions:**

HoLEP in patients with history of OPT for treatment of OC‐PCa is associated with a higher risk of transient urinary incontinence.

## 1 INTRODUCTION

Prostate Cancer (PCa) is the second most common cancer diagnosed in men and a leading cause of cancer‐related morbidity and mortality.^1^, ^2^ Amongst treatment options for organ‐confined PCa (OC‐PCa), radical prostatectomy (RP) offers the advantage of treating cancer and improving Lower Urinary Tract Symptoms (LUTS) associated with an enlarged prostate.^3^ Nevertheless, nearly one‐third of patients with OC‐PCa opt for organ‐preserving treatments (OPT). Radiation therapy (RT)is a commonly used alternative to treat OC‐PCa. It has been observed that LUTS secondary to an enlarged prostate are considered “moderate” or “major problems” by 14% and 18% of patients undergoing external beam radiotherapy (EBRT) and brachytherapy (BT), respectively.^4^ In recent decades, focal therapies such as high‐intensity focused ultrasound (HIFU) have been increasingly used to treat OC‐PCa.^5^ HIFU works by heating tissue to induce coagulative necrosis and is associated with post‐procedure LUTS with up to 10% of patients experiencing urinary retention.^6^, ^7^ Prior literature reports 9.6% of patients required endoscopic intervention for LUTS.^8^ Transurethral resection of prostate (TURP) is commonly performed to relieve bladder outlet obstruction (BOO) and remains the only procedure recommended by the European Association of Urology (EAU) post‐OPT.^6^ While effective in alleviating LUTS, it's limitations include unsuitability for large prostate glands (>80 ml) or patients on anticoagulation.^9^ Prior studies have reported post‐TURP urinary incontinence rates at ≥25% in the context of prior OPT.^10^, ^11^, ^12^ Other surgical options lack sufficient evidence to support their use for this indication.^6^

Holmium Laser Enucleation of the Prostate (HoLEP) is a size‐independent procedure used to treat BOO from Benign Prostatic Hyperplasia (BPH). It is the recommended treatment for patients with LUTS on anticoagulants.^6^, ^9^ While traditionally used for BPH, previous studies have reported the feasibility and safety of HoLEP for LUTS in OC‐PCa patients on active surveillance and advanced PCa patients.^13^, ^14^, ^15^ To the best of our knowledge, there is a lack of literature regarding the use of HoLEP in OC‐PCa patients post‐OPT. In this pilot study, we retrospectively evaluated the safety and efficacy of HoLEP in this unique cohort previously treated with EBRT, BT or collectively grouped as OPT. We also reviewed the literature on outcomes of other procedures to manage these patients.

## 2 METHODS

### 2.1 Patients

We queried a prospectively maintained, IRB‐approved(20180511) database spanning from July 2017 to December 2023 to identify all patients who underwent HoLEP and who had a preoperative diagnosis of OC‐PCa treated by OPT (study group). These patients were matched‐paired (1:2) with BPH patients undergoing HoLEP (control group) for age, prostate size and anticoagulant usage using the nearest‐neighbour method. Exclusion criteria were OC‐PCa patients on active surveillance, locally advanced and/or metastatic disease and those undergoing HoLEP and HIFU in the same setting. Patients with associated neurogenic disorders, urethral stricture, bladder cancer and previous intervention for BPH were excluded from the control group.

### 2.2 Preoperative evaluation

Prostate‐Specific Antigen (PSA) levels were evaluated to assess for recurrence or metastatic disease. Prostate size was measured using either ultrasound (transabdominal or transrectal), computed tomography or magnetic resonance imaging. Evaluation for gross hematuria included urine cytology, computed tomography‐urogram and cystoscopy. Patients with positive urine cultures received culture‐specific antibiotics before HoLEP. Patients were evaluated with the International Prostate Symptom Score (IPSS), peak urinary flow rate (Qmax) and post‐void residual urine (PVR).

### 2.3 Procedure

All procedures were performed by an experienced endourologist with variable trainee involvement using an en‐bloc technique reported previously.^16^ We did not employ early apical release in any of our patients; however modified our technique to preserve some apical tissue in patients from the study group. A 550‐μm laser fibre with Lumenis Pulse™ 120H Holmium Laser System with MOSES™ Technology was used. Enucleation was performed with a 26 Fr laser endoscope (Karl Storz, Tuttlingen, Germany) at energy setting of 2 J and 30 Hz. Morcellation was performed with the VersaCut™ Tissue Morcellator System (Lumenis Inc., Santa Clara, CA, U.S.).

### 2.4 Postoperative management

Our standard post‐treatment pathway included: one‐night observation in the hospital, with a voiding trial on postoperative day 1. All BPH medication was stopped postoperatively, and patients were followed up at 6 weeks, 3 months, 6 months and 12 months with IPSS, Qmax, PVR and for any complications. Urinary incontinence was defined as the presence of involuntary urine leakage of any severity at any time postoperatively.

### 2.5 Outcomes

Perioperative outcomes included operative time, enucleated prostate volume, duration of postoperative catheterization and length of hospital stay. Operative time includes complete time spent by the patient in the operating room, including time spent for additional procedures for accompanying urethral stricture, urolithiasis or bladder tumours. IPSS, Qmax and PVR were evaluated at 3‐months, 6‐months and 12‐months postoperatively. We also obtained a PSA at 3‐months for a new post‐HoLEP nadir. All complications were recorded and included: blood transfusions, failure to void on postoperative day‐1, urinary tract infections (UTIs), ongoing urinary incontinence at 12‐weeks, hematuria, development of urethral stricture and bladder neck stenosis up to 1‐year follow‐up period. In addition, they were assessed using the Clavien‐Dindo classification.^17^ We also reviewed literature on PubMed for all publication on outcomes of surgical management of bladder outlet obstruction in patients with prostate cancer managed with radiation therapy.

### 2.6 Statistical analysis

Continuous variables were reported using mean±standard deviation or median and interquartile range, and were compared using the Wilcoxon signed rank test. Categorical variables were reported using proportions and frequencies and were analysed using Chi‐squared or Fischer's exact test as needed. Analysis was done using RStudio version 2023.09.0(RStudio Inc, Boston, MA). A p‐value<0.05 was considered statistically significant.

## 3 RESULTS

Out of 660 patients who underwent HoLEP, 45 patients were included ‐ 15 in the study group and 30 in the control group. The study group comprised 9 patients post‐EBRT, 7 post‐brachytherapy and 3 post‐whole gland HIFU. Three patients received EBRT and BT. One patient received HIFU and EBRT. The interval between OPT and HoLEP ranged from 3 months to 12 years. None except one patient from study group were on any adjuvant hormonal treatment at the time of HoLEP procedure. Demographics and baseline variables were comparable amongst both groups (Table 1, Table 2). One study group patient was noted to have bladder cancer during HoLEP, which was not detected on preoperative cystoscopy two months prior. It was managed with transurethral resection post‐HoLEP. Two study group patients had accompanying bulbar urethral stricture requiring laser incision (n = 1) and urethral dilatation (n = 1). Another two study group patients had intraoperative diagnosis of prostatic and prostatic urethral calcifications needing laser lithotripsy. Additionally, one patient had both bulbar urethral stricture and prostatic urethral calcifications requiring laser incision and laser lithotripsy, respectively, before HoLEP. Three control group patients had bladder stones requiring subsequent holmium laser cystolithotripsy.

**TABLE 1:** Patient's demographic, baseline information and perioperative outcomes.

Variables	Control group	Study group	P value
N	30	15	
Age (years)	73.5 ± 8.6	71.4 ± 10.7	0.51
BMI	27.0 ± 3.5	26.9 ± 5.0	0.90
Diabetes	23%	50%	0.18
Hypertension	57%	40%	1
**Baseline variables**			
Prostate size (cc)	67.9 ± 29.1	64.4 ± 30.0	0.72
Indication of surgery			
Urine retention	21/30 (70%)	9/15 (60%)	0.73
Hematuria	3/30 (10%)	3/15 (20%)	0.66
Recurrent UTI	6/30 (20%)	3/15 (20%)	1
**Perioperative variables**			
Median Operative Time (mins)	136 [101–201]	119 [92–169]	0.66
Mean Hospital Stay (days)	1.25 ± 0.9	3.0 ± 7.0	0.33
Resected prostate volume	48 [28.2–71.1]	29 [12–43.1]	**0.04**
Histopathology results BPH Prostatitis with radiation atypia Prostate cancer‐ < Gleason 6 Prostate cancer ‐ > Gleason 6	23 (76%) 0 7 0 (0%)	10 (71%) 0 3(21.4%) 1(7.1%)	0.72 ‐ 1 1
**Complications**			
**CD Grade: 1**			
Voiding trial failure on 1st postoperative day (%)	3/30 (10%)	4/14 (28.6%)	0.18
Hematuria	6/30 (20%)	1/13 (7.7%)	0.41
Incontinence reported at 12 weeks *:	4/30 (13.3%)	8/15 (53.3%)	**0.01**
**CD Grade: 2**			
Blood transfusion	1/30 (3.3%)	0/13 (0%)	1
UTI	2/30 (6.7%)	3/14 (21.4%)	0.15
**CD Grade ≥ 3**			
New Onset Urethral stricture	1/30 (3.3%)	1/13 (7.7%)	0.52

(BMI: Body Mass Index; UTI: Urinary Tract Infection; CD: Clavien‐Dindo Grade).

**TABLE 2:** Follow‐up outcomes.

Time	Control group	Study group	P value
***IPSS***			
Baseline	15 [11–22]	16 [13–21]	0.81
3 months	2 [0–6]	3.5 [2–7]	0.13
6 months	0 [0–1]	3 [2–5]	**0.02**
12 months	0 [0–4]	4 [1–6]	0.19
Qmax (mL/s)			
Baseline	10.2 ± 6.7	9.4 ± 6.1	0.59
3 months	17.1 ± 12.2	20.9 ± 18.1	0.56
6 months	23.5 ± 18.3	28 ± 0.5	0.28
12 months	20.9 ± 13.1	21.3 ± 7.1	0.99
PVR (mL)			
Baseline	174 [48–290]	130 [97–293]	0.49
3 months	23 [5–38]	12 [5–31]	0.71
6 months	12 [2–46]	0 [0–24]	0.47
12‐months	4 [0–26]	11 [0–43]	0.89
PSA (ng/mL)			
Baseline	2.9 [1.8–5.7]	1.0 [0.1–5.6]	0. 08
3 months	0.3 [0.20–0.70]	0.27 [0.11–0.6]	0.87

(IPSS: International Prostate Symptom Score; PVR: Post‐void residual volume; Qmax: Maximum urinary flow rate. Median [IQR ‐Interquartile range 25th to 75th]).

Operative times were comparable between the study group and control groups. Overall, we noticed greater difficulty in identifying and maintaining the enucleation plane in the study group patients. Histopathology of enucleated tissue revealed Gleason 9 prostate cancer in only 1 patient (7.1%) and Gleason 6 cancer in 3 (21.4%) patients (Table 1). All seven patients in control group diagnosed with prostate cancer had Gleason 6 cancer. All patients with Gleason 6 prostate cancer in either group were on active surveillance at the time of their last follow‐up. The patient with Gleason 9 cancer, aged 83 years, had negative metastatic work‐up and was initially treated with androgen deprivation therapy. Unfortunately, he died 2 years after HoLEP due to myocardial infarct. Amongst perioperative outcomes, the volume of enucleated prostatic tissue was lower in the study group compared to the control at median (IQR) of 29[12–43.1] vs 48[28.2–71.1] (p < 0.04) (Table 1). Postoperative voiding outcome was clinically comparable amongst both groups (Table 2).

Regarding complications, the rate of urinary incontinence was higher in the study group compared to the control group at 53.3% (8/15) vs 13.3% (4/30), respectively (p < 0.01) (Table 1). At 1‐year follow‐up, two patients (13.3%) continued to experience incontinence in the study group compared to none in the control group. Both patients with preexisting bulbar urethral stricture in the study group experienced recurrence at 3 months and 11 months postoperatively. One received Optilume urethral dilatation, and another was managed conservatively. These were the patients who continued to have persistent stress incontinence at 1 year.

The outcomes of surgical management of bladder outlet obstruction in patients with prostate cancer managed with radiation therapy is summarized in Table 3.

**TABLE 3:** Literature review on outcomes of surgical management of bladder outlet obstruction in patients with prostate cancer managed with radiation therapy.

Study (published year, country) type of study.	N	OPT type	Prostate size mean or median (range)	Indication for BOO surgery	Median duration since OPT [range] (month) weight of resected tissue (gm)	Duration of follow upMean/median (range)	Postop. Incontinence (%)	Comments
**TURP**								
Holzman et al (1991, USA) RS^18^	44	Combine EBRT & BT	NA	BOO: 44/44.	NA NA	NA	27%	All patients had baseline clinical Stage 3 PCa and developed local recurrence. 44 patients needed 66 TURP.
Patel et al (1997, USA) RS^19^	7	6: EBRT 1: combine EBRT & BT	NA	LUTS	NA Mean 22 [2–81]	2.9 year (3 m‐ 4.5 yr)	0	Minimal TURP Baseline T3–4 disease 1 patient‐ urge incontinence 3/7 had cancer on HPE
Hu and Wallner (1998, USA) RS^10^	10	BT	62 cc	UO: 10/10.	NA NA	Median 3 yr [1.3–4.4]	70	Baseline T1‐T2 PCa. Incontinence does not depend on amount of tissue resected, or time between brachytherapy and TURP/TUIP
Hirschberg and Klotz et al (1998, Canada) RS^20^	16	EBRT	NA	LUTS	5.0 [1 to 81]. NA	median 5.0 m (1 to 81)	19	Retention: 6.25%
Gelblum et al (1999, USA) RS^21^	28	BT	NA	LUTS	NA NA	NA	17	
Koutrovelis et al (2003, USA) RS^22^	11	BT	NA	NA	12 to 48 NA	NA	27.3	
Flam et al (2004, France) RS^23^	19	BT	40 (25–50)	UR: 11/19. LUTS: 8/19.	7 [6–41] Median 8 (2 to 19)	Median 9 m (1–37)	0	Minimal TURP Dysuria & retention: 5.26% Recommend 6‐month interval after BT for TURP HPE‐ all Benign
Kollmeier et al (2005, USA) RS^24^	38	BT	NA	UR: 17/38. LUTS 17/38. hematuria: 4/38	9 [1–62] NA	NA	18	Minimal TURP Suggest longer duration > 2 year between RT and TURP may increase risk of incontinence.
Mock et al (2013, USA) RS^11^	79	BT	46.7 (12–84)	UR:11/79 or LUTS: 68/79	14.8 NA	48 m (0 to 186 m)	25.3	
Abelson et al (2014, USA) RS^25^	40	BT	Median 53 (26–76)	UR: 36/40. Hematuria: 2/40. UTI: 2/40	20.9 (12.6,33.9) NA	Mean 5.4 yr	40	Retreatment ‐ 47.5%
	18	EBRT	NA	UO: 16. Hematuria:1 UTI: 1	55 (22,80) NA	Mean 5.4 yr	39	Additional surgical intervention 28%
**Photo‐selective vaporization of prostate (Green‐light prostatectomy)**								
No et al (2013, USA) RS^26^	12	6‐ BT 6‐ ERBT	Median 34 (30.1, 60.0)	Chronic UR or LUTS	NA NA	Median 22.9 (13.4, 41.7)	0	Catheter dependent −8.33% No relation of duration between radiation and PVP and incontinence.
Abelson et al (2014, USA) RS^25^	20	BT	Median 51 (41, 64)	UR: 19/20. UTI: 1/20	NA NA	Mean 6.7 yr	35	Additional surgical intervention ‐ 50%
	10	EBRT	NA	UO: 9. Hematuria:1	NA NA	Mean 6.7 yr	39	Additional surgical intervention ‐ 28%
**HoLEP**								
Current study (2024, USA) RS	16	EBRT, BT, HIFU	60.5 (47.9–96.5)	BOO: 9/15; Hematuria: 3/15 Recurrent UTI: 3/15	3 to 244 Median 29 [17–49.3]	Range 3 months to 12 years	Incontinence‐ 8/15 (53.3%)	Urine retention‐ 4/14 (28.6%)

(PCa – Prostate Cancer; RS – Retrospective Study; EBRT – External Beam Radiation Therapy; BT – BrachyTherapy; HIFU – High Intensity Focused Ultrasound; TURP – TransUrethral Resection of Prostate; TUIP – TransUrethral Incision of Prostate; PVP – Photoselective Vaporization of Prostate; BNS – Bladder Neck Stenosis; NA – Not available; BOO – Bladder Outlet Obstruction; LUTS – Lower Urinary Tract Symptoms; UO: Urinary obstruction; HPE – HistoPathological Examination).

## 4 DISCUSSION

In this retrospective study, we present the first 1:2 matched comparison between patients who underwent HoLEP post‐OPT for OC‐PCa and those who underwent HoLEP for BPH. Overall, the OC‐PCa group exhibited a higher incidence of associated urethral stricture, prostatic urethral calcification and bladder tumours. Despite these, HoLEP resulted in similar improvements in voiding parameters, although the OC‐PCa group was at higher risk for postoperative urinary incontinence. The literature on surgical management of enlarged prostate after previous RT is sparse, and most of the outcomes are reported after TURP, with two studies employing photoselective vaporization of prostate (PVP) (Table 3).

Historically, RT was employed to treat clinically T3/T4 PCa. Gibbons et al reported that 8.1% of patients treated with EBRT for clinical stage‐C PCa required operation at mean of 46 month (range 4–12 months). Of these 7% were for local cancer recurrence and 1.1% were for BPH.^27^ Regarding the interval between initial RT and prostate surgery, Abelson et al noted that patients needed surgery much earlier after BT than EBRT with the median time being 20.2 months(range, 14.6–27.6) and 53.3 months (range, 27.5–53.3), respectively.^25^ However, the time interval between BT and/or EBRT and the need for BOO surgery varied considerably (Table 3). Similarly to prior reported literature, indications for surgery post‐OPT in our study were urine retention (60%), recurrent gross hematuria (20%) and recurrent UTI (20%).

Overall, 28.6% of study group patients had evidence of PCa on histopathological examination of prostate tissue after enucleation. This might explain the differences in the baseline PSA level. In older series, more patients were diagnosed with cancer based on tissue removed during TURP than in our study, but this is likely because their cohort had clinically locally advanced PCa and not OC‐PCa as in our study.^18^ Flam et al found no evidence of cancer on TURP specimens.^23^

Although baseline prostate volume was similar between groups, the enucleated volume was significantly lower in the OC‐PCa arm compared to the BPH arm. Operative times were comparable between the study group and control groups. No et al reported significantly shorter median operative times of 48 minutes with PVP procedures.^26^ However, their baseline prostate size was much smaller with median 34 ml (IQR 30.1–60) as compared to a mean 64.4 ml ± 30.0 in our study. Additionally, 6 study group patients needed additional surgery for bladder tumours, urethral strictures or prostatic urethral calcifications, adding to operative time in our study. None of the studies reporting TURP outcomes for patients with PCa post‐RT reported operation times.^10^, ^11^, ^19^, ^20^, ^21^, ^22^, ^23^, ^24^, ^25^, ^28^ Enucleated prostate volume was higher in the current study compared to previously reported volumes since most of the previous studies described a “minimal” resection of 8–10 g.^11^, ^23^, ^24^ This, along with additional pathological findings such as calculi and strictures requiring intervention in the control group, may explain the comparable surgical times despite the smaller tissue volume removed in the OC‐PCa group. Nadir PSA levels at 3 months post‐HoLEP were comparable between groups, suggesting similar completeness of adenomectomy. However, nadir PSA may not be a reliable indicator of enucleation completeness in prostate cancer patients. One control group patient was receiving hormonal therapy at the time of HoLEP, which may have influenced PSA levels. Additionally, prior use of androgen deprivation therapy could have confounded PSA results.

We report excellent improvement in voiding function in both groups. Similar to our series, Liu et al reported an improvement in mean IPSS from 14.58points to 5.21points, mean Qmax from 7.93 ml/s to 16.78 ml/s and mean PVR from 132.43 ml to 35.84 ml post‐TURP in patients who received BT for OC‐PCa.^29^ Patel et al reported similar improvement in Qmax from median 4 to 14.8 cc/s amongst patients who underwent TURP after EBRT for PCa.^19^ After PVP, No et al found that IPSS improved only from a baseline median of 15points to 10points at 1‐year follow‐up. However, they reported improvements in Qmax and PVR from baseline median 4 ml/s to 15.9 at 1‐year and baseline median 200 ml to 5 ml at 1‐year, respectively.^26^ We believe that radiotherapy inherently might cause bladder irritability, which may be reflected in the higher IPSS in study group patients in our series. Evidence on the efficacy of TURP with respect to voiding parameters improvement in OC‐PCa patients treated with HIFU is lacking.

Since the initial report by Kaufman et al of an increase in urinary incontinence after RT as a postoperative adjuvant or preceding salvage prostatectomy for PCa, it is perceived that patients with prior RT for PCa are more likely to experience postoperative incontinence.^30^ It is hypothesized that RT might reduce bladder compliance, cause urethral or periurethral fibrosis, which is shown to alter elasticity of the external urethral sphincter. It is likely that postoperative SUI is secondary to external sphincter fibrosis after RT that sometimes result in lead pipe membranous urethra, which could fracture with the introduction of resectoscope sheath, worsened by torquing of resectoscope. Authors previously hypothesized that the addition of TURP possibly alters both the proximal and distal sphincter mechanism resulting in increased incontinence.^30^, ^31^ In the present series, we noted that HoLEP was associated with a 53.3% rate of urinary incontinence at 12‐weeks postoperatively in OC‐PCa patients compared to 13.3% patients in the BPH arm. Two study group patients who continued to experience incontinence at 1‐year follow‐up, notably had urethral strictures detected incidentally at the time of HoLEP. The risk of incontinence reported in the literature varied significantly from 0% to 70% (Table 3). A “minimal” TURP strategy was recommended to help to avoid incontinence after TURP in post‐brachytherapy patients.^19^, ^23^ However similar minimal TURP resulted in 18% incontinence.^24^ No et al reported no urinary incontinence in the 30‐day postoperative period after PVP.^26^ These authors performed limited vaporization and photocoagulation at the apex that might be responsible for preventing postoperative urinary incontinence. Furthermore, they used a 24F scope (versus 26F in the current study) which may possibly result in decreased mechanical manipulation of the post‐OPT external sphincter with decreased elasticity. Based on our findings, physicians should counsel patients who are considering HoLEP for post‐OPT LUTS that there may be an increased risk of transient urinary incontinence, which is similar to what was seen post‐TURP.^32^ Patients should also be counselled regarding the impact for higher rates of permanent or long‐term incontinence as it can have significant impact on quality of life especially if they are already suffering from significant storage and irritative bladder symptoms. It is arguable that an alternative modified HoLEP template to preserve apical tissue as done by No et al might be helpful in reducing postoperative incontinence. However, this strategy should be balanced with possible need for additional surgery for recurrent/persistent obstruction. Abelson et al compared the outcomes of TURP and PVP in irradiated PCa patients.^25^ Differences found in the efficacy, rates of repeat procedures and rates of urinary incontinence between the two procedures in these patients were not statistically significant. They noted that up to 50% of patients would need additional procedures for obstruction regardless of TURP or PVP procedure.^25^ In our series no patient needed repeat procedures for BPH at up to 1‐year follow‐up. However excessive removal of prostate tissue in post prostate RT scenario might be theoretically associated with increased risk of rectal fistula. Fortunately, none of the patients in our study had this complication.

Patients with prior OPT for PCa tend to have higher rates of urethral strictures and prostatic calcifications, which can hamper the postoperative outcomes of outlet procedures like TURP or HoLEP.^33^, ^34^, ^35^ We also found a patient with incidental bladder cancer, possibly secondary to exposure to radiation for prostate cancer.^34^ Due to the high possibility of associated lower tract urological anomalies, we also suggest conducting a screening cystoscopy for all patients who have previously undergone OPT for OC‐PCa for better preoperative planning and patient counselling.

While data comparing the outcomes of patients undergoing BOO surgeries before or after OPT is scarce, it is generally accepted that BOO surgery done pre‐OPT in those with pre‐existing bothersome LUTS (expected to worsen post‐OPT) is safer than doing BOO surgery post‐OPT.^36^, ^37^ Delgado et al^38^assessed the functional outcomes of patients who underwent HoLEP prior to HIFU within the same session to prevent exacerbation of bladder outlet obstruction LUTS post‐HIFU for OC‐PCa. They reported lower absolute rates of adverse events (compared to the current study) – urinary retention at 10% (versus 60%), urinary incontinence at <20% (versus 53.3%) and urethral strictures at 3.03% (versus 7.7%). This difference might be due to inclusion of radiotherapy patients in our group. It is possible that patients with prior radiation therapy might have inferior outcome as compared with patients with prior HIFU for management of OC‐PCa. Due to the relatively small number of patients, we were not able to do sub‐group analysis of patient undergoing RT or HIFU as initial treatment for OC‐PCa. Although it is preferred to relieve bladder outlet obstruction before OPT to avoid exacerbation, a direct comparison of patients undergoing bladder outlet procedures before and after OPT would be informative.

Limitations of our study include small sample size, retrospective nature and short follow‐up. Due to the small number of patients, we included patients with various sub‐types of radiotherapy and whole‐gland HIFU in our study group. It is likely that the outcome of HoLEP in post‐radiation therapy patients and those with prior whole gland HIFU might differ. Assessment of completion of enucleation in post‐OPT group may not be the best done by drop in PSA levels due to pre‐existing low levels from OPT and androgen deprivation therapy that they may have received for OC‐PCa. We lacked data on post‐HoLEP prostate volume to confirm completeness of enucleation. The results from this study may not be generalizable as all surgeries were performed or supervised by a single experienced surgeon. Despite these, the present study is the first to our knowledge to evaluate the safety and efficacy of HoLEP in OC‐PCa post‐OPT. Our study and related review of the literature might help in preoperative counselling of these complex patients.

## AUTHOR CONTRIBUTIONS

Protocol/project development: HNS, JK, RM. Data collection or management: JGP, JCD, AR, MM, AR, AK. Data analysis: AB, RST, HNS. Manuscript writing/editing: RST, AB, JGP, JCD, AR, MM, AK, AR, JK, RM, HNS.

## CONFLICT OF INTEREST STATEMENT

The authors have no conflicts of interest.

## STATEMENTS AND DECLARATIONS

No funding was received to conduct this research.
